# Impaired Fasting Glucose and Diabetes as Predictors for Radial Artery Calcification in End Stage Renal Disease Patients

**DOI:** 10.1155/2013/969038

**Published:** 2013-12-18

**Authors:** Katarzyna Janda, Marcin Krzanowski, Mariusz Gajda, Paulina Dumnicka, Danuta Fedak, Grzegorz J. Lis, Piotr Jaśkowski, Jan A. Litwin, Władysław Sułowicz

**Affiliations:** ^1^Chair and Department of Nephrology, Jagiellonian University Medical College, 31-501 Krakow, Poland; ^2^Chair and Department of Histology, Jagiellonian University Medical College, 31-501 Krakow, Poland; ^3^Department of Medical Diagnostics, Jagiellonian University Medical College, 31-501 Krakow, Poland; ^4^Chair of Clinical Biochemistry, Jagiellonian University Medical College, 31-501 Krakow, Poland

## Abstract

*Objective.* The objective of the study was to assess the relationship between selected clinical and biochemical parameters of end stage renal disease (ESRD) patients and arterial calcification. *Materials and Methods.* The study comprised 59 stage 5 chronic kidney disease patients (36 hemodialyzed and 23 predialysis). The examined parameters included common carotid artery intima-media thickness (CCA-IMT), BMI, incidence of diabetes and impaired fasting glucose (IFG), dyslipidemia, hypertension, and 3-year mortality. Plasma levels asymmetric dimethylarginine (ADMA), osteopontin (OPN), osteoprotegerin (OPG), and osteocalcin (OC) were also measured. Fragments of radial artery obtained during creation of hemodialysis access were stained for calcifications using von Kossa method and alizarin red. *Results.* Calcification of radial artery was significantly associated with higher prevalence of IFG and diabetes (*P* = 0.0004) and older age (*P* = 0.003), as well as higher OPG (*P* = 0.014) and ADMA concentrations (*P* = 0.022). Fasting glucose >5.6 mmol/l (IFG and diabetes) significantly predicted vascular calcification in multiple logistic regression. The calcification was also associated with higher CCA-IMT (*P* = 0.006) and mortality (*P* = 0.004; OR for death 5.39 [1.20–24.1] after adjustment for dialysis status and age). *Conclusion.* Combination of renal insufficiency and hyperglycemic conditions exerts a synergistic effect on vascular calcification and increases the risk of death.

## 1. Introduction

Vascular calcification is an active process similar to the mineralization that occurs in bone [1, 2]. Vascular smooth muscle cells undergo phenotypic differentiation into osteoblast-like or chondroblast-like cells and they synthesize calcification regulating proteins and matrix components typically found in bone and in cartilage [3, 4]. Calcification of the arterial media, observed even in small vessels (Mönckeberg's calcification), is common in uremic patients and seems to be less associated with inflammation as compared to intimal mineralization typical for atherosclerosis [5, 6].

Vascular mineralization advances with age and is intensified in diabetes, dyslipidemia, chronic kidney disease, and hypertension. In newly treated hemodialysis and peritoneal dialysis patients, diabetes, dialysis duration, and the previous presence of aortic arch calcification (AAC) accelerate further progression of AAC (an important risk factor for cardiovascular complications) [7]. Hyperinsulinemia and insulin resistance (clinical signs of type 2 diabetes) are positively correlated with the arterial calcification [8, 9]. Arterial medial calcifications often occur also in diabetic individuals as a component of the diabetic macroangiopathy. In the animal model, insulin resistance induced in rats by fructose feeding resulted in increased aortic calcium deposition, elevated calcium-phosphate index, and local hyperplastic changes in the aortic media [10].

Blood vessels obtained from end stage renal disease (ESRD) patients were often studied by various histological techniques to assess vascular calcification. They were collected during renal transplantation (epigastric or iliac arteries) or by peripheral arterial biopsy (radial artery) [3, 11, 12].

In the present study we used small samples (otherwise routinely discarded) of radial arterial walls obtained during creation of arteriovenous fistula for hemodialysis access. The study was aimed at investigating the relationship between selected clinical and biochemical parameters, with special emphasis on diabetes markers, and the level of histologically assessed radial artery calcification in end stage renal disease patients.

## 2. Materials and Methods

### 2.1. Patients

The study population consisted of 59 patients (38 males, 21 female; mean age at the beginning of the study 61 ± 16 yrs), including 36 on maintenance hemodialysis (HD) and 23 on predialysis (stage 5 of CKD). The study was approved by the Bioethics Committee of the Jagiellonian University and all patients signed an informed consent for their participation. The data on mortality was collected over a period of three years. During this period, all the patients were treated at the Department of Nephrology, Jagiellonian University Hospital. The mortality data, including the causes of death, was based on the patients' records.

### 2.2. Laboratory Tests

In all patients, selected biochemical parameters were assessed: serum concentrations of total cholesterol, HDL-cholesterol, LDL-cholesterol, triglycerides (TG), serum creatinine, peripheral blood counts, albumin, glucose, intact parathyroid hormone (iPTH), total calcium (Ca) and phosphate (Pi), high sensitive C-reactive protein (hsCRP), asymmetric dimethylarginine (ADMA), osteopontin (OPN), osteoprotegerin (OPG), and osteocalcin (OC). Homeostasis Model of Assessment-Insulin Resistance (HOMA-IR) was calculated by application of the international formula: fasting insulin (*μ*IU/mL) × fasting glucose (mmol/L)/22.5 [13].

Routine biochemical tests were carried out using automatic biochemical analyzers: Hitachi 917 (Hitachi, Japan) and Modular P (Roche Diagnostics, Mannheim, Germany). Concentrations of hsCRP were measured using immunonephelometric method on Nephelometer BN II (Siemens Healthcare Diagnostics, Germany). Hematological parameters were measured using Sysmex XE 2100 Hematological Analyzer (Sysmex Corp., Japan).

OPN, OC, OPG, and ADMA were determined using ELISA microplate immunoassays and ELX808 automatic reader (BIO-TEK Instruments, Inc., Vermont, USA). The following kits of reagents were applied: OPN (R&D Systems), OC (METRA, Germany), OPG (QUIDEL, BioVendor, Czech Republic), and ADMA (Immundiagnostik, Germany).

The mean arterial pressure (MAP) was calculated according to the formula: MAP = DBP + 1/3(SBP-DBP), where SBP is systolic blood pressure and DBP is diastolic blood pressure.

The intima-media thickness of the common carotid artery trunk (CCA-IMT) was assessed by ultrasonography (B presentation, Acuson 128 XP/10 apparatus equipped with a linear head at 5/7 MHz). The measurements were performed bilaterally at 0.5 cm and 2 cm below the division of the common carotid artery during diastolic phase of the heart cycle. The results were expressed as the arithmetic means of the values obtained for the left and right arteries.

### 2.3. Histology

Fragments of radial artery, approx. 5 × 2 mm in size, were collected during the first creation of arteriovenous fistula for hemodialysis access. The samples were immediately immersed in 10% phosphate-buffered formalin and fixed overnight and then rinsed in PBS and soaked in 30% sucrose. The material was snap-frozen and tissue blocks were positioned in a cryostat for cutting sections in a plane encompassing the entire thickness of the vascular wall. Serial 10 *μ*m-thick cryosections were cut and thaw-mounted on poly-L-lysine coated slides. Sections were stained routinely with Mayer's haematoxylin and eosin (HE), with von Kossa method and with alizarin red. The stained sections were examined using an Olympus BX-50 microscope (Olympus, Tokyo, Japan) in brightfield mode and images were registered using Olympus DP-71 digital CCD camera controlled by Olympus AnalySIS FIVE software. The advancement of vascular calcification was semiquantitatively evaluated in von Kossa and alizarin red-stained sections by two independent observers. The degree of mineralization was classified according to the following scale: 0: no mineral content, 1: a few small dispersed concretions, 2: numerous small dispersed concretions, 3: larger granular concretions, and 4: large areas occupied by fused mineral deposits. The reproducibility of the morphological analysis was confirmed by Bland-Altman method and by calculating intraclass correlation coefficient (ICC) which was 0.88.

### 2.4. Statistical Methods

The number of patients (percentage of the group) was reported for categorical variables and mean ± standard deviation or median (lower-upper quartile) for continuous variables, depending on distribution. The Shapiro-Wilk test was used to assess normality. Contingency tables were analyzed with Pearson chi-squared test. Student *t*-test or Mann-Whitney test was used for simple comparisons between the groups. Multiple logistic regression models were constructed using the variables that differed significantly between the groups in simple comparisons and/or predefined sets of confounders, as pointed in the results. Odds ratios (OR) for 1 unit increase with 95% confidence intervals (95% CI) being reported, unless otherwise stated. All tests were two-tailed and the results were considered statistically significant at *P* ≤ 0.05. Statistica 10 software (StatSoft, Tulsa, OK, USA) was used for computations.

## 3. Results

### 3.1. Histology

**Figure 1:**
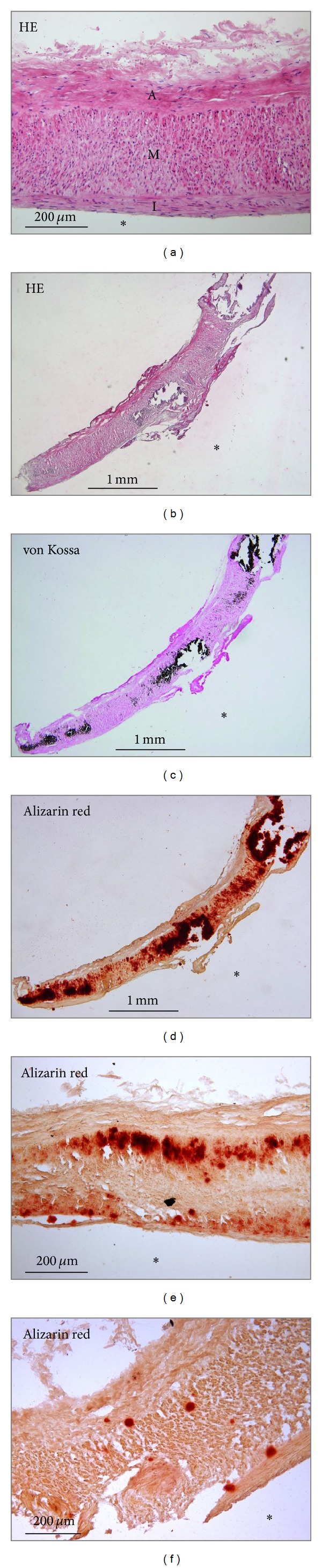
Histology of the radial artery samples. (a) Morphology of the routinely (HE) stained artery showing intimal thickening (I) and no mineral content. I: intima; M: media; A: adventitia. (b)–(d) Serial sections of the entire sample of the arterial wall demonstrating very advanced calcification (grade 4) stained with HE (b), von Kossa (c), and alizarin red (d). (e) Large granular calcifications (grade 3) localized on both sides of the media (alizarin red staining). (f) Small (grade 2) calcifications in the media (alizarin red staining). *Lumen of the vessel.

Routine histology (HE) showed general morphology of the radial artery, with intimal thickening in the vast majority of the examined specimens (Figure 1(a)). Basophilic deposits were visible in arterial wall in cases of very intense calcification (Figure 1(b)). The preliminary comparison of two staining methods used for the assessment of the mineralization degree showed that von Kossa method was less sensitive; thus we employed alizarin red staining for further analysis and for correlation of the vascular calcification level with clinical and biochemical data.

Morphologically, mineral deposits were found in all layers of the arterial wall but they were most frequently localized in the media (Figures 1(b)–1(d)). In less advanced lesions, deposits were preferentially located close to the outer and inner elastic laminae (Figure 1(e)). Some deposits were fine and dispersed (Figure 1(f)), while others occupied larger areas and in the most advanced cases even the entire thickness of the media (Figures 1(b)–1(d)). Only very scanty mineral deposits were occasionally seen in the vascular intima.

**Figure 2:**
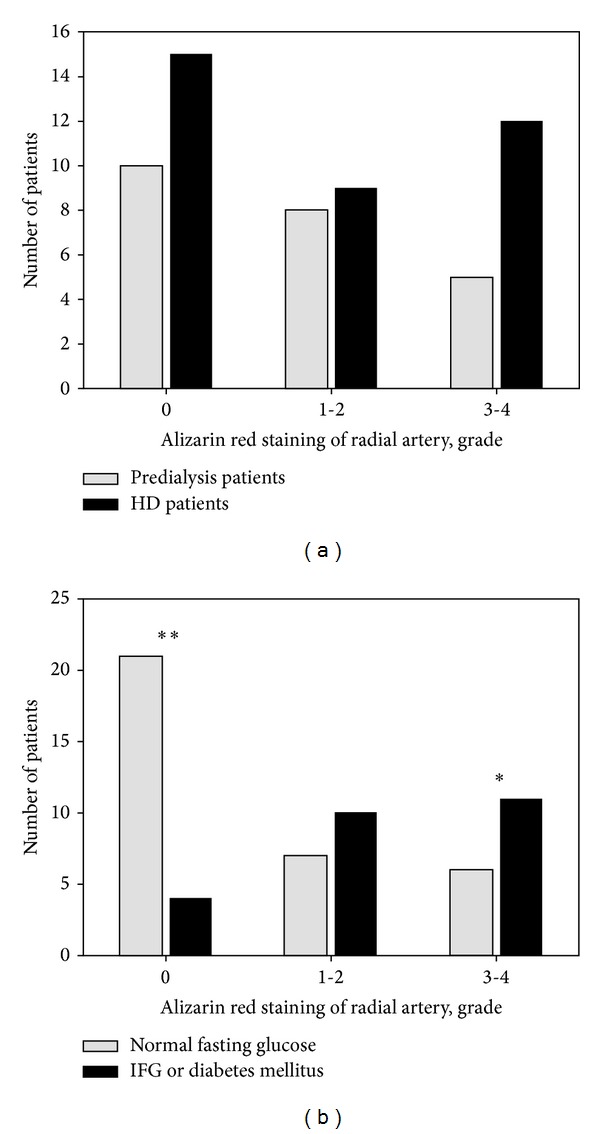
Advancement of vascular calcification as assessed by alizarin red staining in radial arteries: 0: no mineral deposits in arterial wall, 1-2: mild calcification, and 3-4: advanced calcification. (a) Calcification in predialysis patients versus HD patients (*P* = 0.6 in chi-squared test). (b) Calcification in patients with normal fasting glucose (<5.6 mmol/L) versus IFG (prediabetes) or diabetes (*P* = 0.002 in chi-squared test; *P* = 0.004 (**) and *P* = 0.03 (*) in post hoc tests). IFG: impaired fasting glucose.

Among 59 radial artery samples examined histologically, 34 showed positive alizarin red staining indicative of the calcification process. The proportion of samples with arterial wall mineralization (Table 1) as well as its advancement (Figure 2(a)) did not differ between HD and predialysis patients (*P* = 0.6). In further analysis, HD patients were studied together with predialysis patients; nevertheless all multiple regression models were adjusted for HD status.

### 3.2. Biochemical and Clinical Data

Table 1 summarizes differences in clinical and biochemical parameters between the groups with and without calcifications as assessed by alizarin red staining of radial artery. Vascular calcification was associated with higher age of patients, higher glucose, and diabetes. MAP was slightly lower in patients with calcifications and the levels of ADMA and OPG were higher in this group.

Clinical criteria of the metabolic syndrome [14, 15] were compared between patients with and without vascular calcifications in radial artery (Table 2). Among patients with vascular calcifications, the number of individuals with fasting glucose level above 5.6 mmol/L, that is, patients with IFG (prediabetes) [16] and diabetes, was significantly higher (*P* = 0.0004). Moreover, vascular calcifications were more severe in the group of patients with IFG and diabetes (Figure 2(b)). Other criteria of the metabolic syndrome did not differ between the groups with or without calcifications.

The association of radial artery calcifications with IFG and diabetes was further confirmed by multiple logistic regression (Table 3). Three models were constructed, containing age and fasting glucose > 5.6 mmol/L as independent variables: the first one was adjusted for gender, HD status, and Ca × Pi product, and the second was additionally adjusted for dyslipidemia, hypertension, high BMI, and hsCRP. The third model, adjusted as the first one, included other variables that were significantly associated with arterial calcification in simple comparisons, that is, MAP, ADMA, and OPG. Fasting glucose > 5.6 mmol/L was the only variable independently associated with the vascular calcifications in all three models. The results were similar when diabetes was substituted for increased fasting glucose level in the models: OR = 17.2 (1.79–166); *P* = 0.011 in model 1; OR = 25.4 (1.74–372); *P* = 0.015 in model 2; OR = 24.6 (1.45–419); *P* = 0.022 in model 3.

Patients with calcifications in radial arteries presented higher CCA-IMT (Table 1). The relation between CCA-IMT and histologically detected vascular calcification remained significant after adjustment for dialysis status, gender, Ca × Pi, hsCRP, and the presence of metabolic syndrome (OR for vascular calcification 2.31 per 0.1 mm increase in CCA-IMT; 95% CI 1.18–4.52; *P* = 0.011) but not after adjustment for age. CCA-IMT was significantly higher in patients with IFG and diabetes (0.98 ± 0.13 versus 0.88 ± 0.15 mm; *P* = 0.007).

Sixteen patients with calcifications in the radial artery (47%) died during 3 years of the followup, while in the group without calcifications the mortality was lower: 3 deaths (12%). All except 3 deaths occurred due to cardiovascular causes (Table 1). Vascular calcification was significantly associated with patients' mortality in simple analysis (*P* = 0.004) and after adjustment for HD status and age (OR for death 5.39; 95% CI 1.20–24.1; *P* = 0.024).

## 4. Discussion

This study presents a comprehensive comparison of biochemical and clinical data with the calcification status assessed histologically in the peripheral arteries of ESRD patients. Biopsies of radial artery collected during the creation of vascular access for hemodialysis were used previously by other authors to study calcification. However, in that study, the surgical anastomosis was performed in an end-to-end fashion; thus a sample encompassing the entire circumference of the artery could be excised and used for further analysis, allowing for a more reliable assessment of the calcification extent in the arterial wall [12].

In the study mentioned above, the authors found mineral deposits in 37% of the examined radial arteries. After adopting stringent morphological criteria to include even the finest calcifications and using more sensitive alizarin red staining for calcium detection we found arterial calcification in 57% of cases.

Calcification can develop in two distinct layers of the artery: intima and media [17]. Intimal calcification occurs in advanced atherosclerotic lesions and is associated with lipid accumulation and infiltration of the inflammatory cells, such as macrophages and T cells. Medial arterial calcification (MAC) displays features very similar to those of physiological calcification in bone [18]. MAC develops independently of atherosclerosis and is a commonly observed pathology in diabetes, ESRD, and ageing [17].

The present study revealed a significant association of arterial medial calcifications with impaired fasting glucose (IFG, prediabetes) and diabetes but not with other criteria of the metabolic syndrome including overweight. Our results are in accordance with the study of Lim et al. [19] demonstrating the relationship between anthropometric parameters, metabolic profiles, and coronary artery calcium scoring (CACS). Subjects with IFG or diabetes had higher CACS and more advanced coronary stenosis than normal subjects. Moreover, several studies confirmed that fasting plasma glucose is a better independent determinant of the progression of coronary artery calcification than the other metabolic syndrome risk factors [20–22].

The above mentioned papers presented a relationship between hyperglycemia and vascular calcification based on noninvasive imaging of blood vessels. In our study, this relationship was analyzed for the first time using histologically examined samples of peripheral arteries.

Hyperglycemia is an established risk factor for cardiovascular disease. Our study showed that fasting hyperglycemia, mostly associated with type 2 diabetes, was the only significant predictor of vascular calcifications in ESRD patients. Consistently, type 2 diabetes was associated with more severe calcification. Recent evidence suggests that medial calcification in diabetes is an active, cell-mediated process, similar to that observed in patients with end stage renal disease [23, 24].

Vascular calcifications and atherosclerosis are frequent in patients with ESRD and they are associated with increased cardiovascular morbidity [25]. Coronary artery calcification (CAC) was found in 70.2% of dialysis patients and was significantly associated with CCA-IMT and the thickness of atherosclerotic plaques [26]. These results indicate that both, medial calcification and atherosclerotic lesions, frequently coexist in patients with ESRD and that CCA-IMT, increased in patients with calcifications examined in this study, may serve as a surrogate marker of vascular calcification.

The mechanisms responsible for vascular calcification include inflammation and oxidative stress, as well as bone and mineral metabolism disturbances. In our study higher ADMA and OPG levels were associated with vascular calcification. High ADMA levels are associated with endothelial dysfunction and cardiovascular damage [27]. Serum levels of ADMA in chronic kidney disease increase due to its defective inactivation and excretion. Coen at al. [28] postulated that ADMA may play a role in the pathogenesis of vascular calcification in dialysis patients. Increased serum OPG is associated with type 2 diabetes, chronic kidney disease, and the severity of vascular calcification and coronary artery disease [29–31]. It could represent a compensatory mechanism for vascular damage, also showing a protective effect against vascular calcification [32, 33].

Arterial calcification was associated with higher mortality in ESRD patients. According to our knowledge, the relationship between vascular calcification assessed histologically and the long-term mortality in chronic kidney disease patients has not yet been studied. Ogawa et al. [34] examined the effect of CT-assessed aortic arch calcification on mortality in the 401 hemodialysis patients during 4-year follow-up period and demonstrated that cardiovascular mortality was significantly higher in patients with calcification. In our study, employing a different assessment model (radial artery and histology) this effect of arterial calcification on mortality in ESRD patients has been confirmed.

## 5. Conclusions

Small samples of radial artery obtained during creation of vascular access for hemodialysis may successfully serve as source of the material for histological assessment of vascular mineralization. In end stage renal disease patients, impaired fasting glucose (prediabetes) and diabetes predict vascular calcification which is significantly associated with higher mortality. These results indicate that combination of renal insufficiency and hyperglycemic conditions exerts a synergistic effect on vascular calcification and increases the risk of death.

## Figures and Tables

**Table 1:** Differences in clinical parameters between patients with and without vascular calcifications as assessed by alizarin red staining of the radial artery.

	Patients with vascular calcifications (grades 1–4, *N* = 34)	Patients without vascular calcifications (grades 0, *N* = 25)	*P*
Male gender, *N* (%)	23 (68%)	15 (60%)	NS
HD treatment, *N* (%)	21 (62%)	15 (60%)	NS
HD duration, months^a^	10 (3–36)	6 (1–38)	NS
Hemoglobin, g/dL	10.7 ± 1.6	11.5 ± 1.9	NS
Albumin, g/L	40.3 ± 4.2	41.5 ± 6.1	NS
Age, years	66 ± 15	54 ± 14	0.003
Active smoking, *N* (%)	10 (29%)	7 (28%)	NS
BMI, kg/m^2^	26.1 ± 5.6	26.4 ± 6.1	NS
Diabetes, *N* (%)	18 (53%)	1 (4%)	<0.0001
Type 1 diabetes, *N* (%)	2 (6%)	0	NS
Type 2 diabetes, *N* (%)	16 (47%)	1 (4%)	0.0002
IFG, *N* (%)	3 (9%)	3 (12%)	NS
SBP, mmHg	138 ± 19	146 ± 18	NS
DBP, mmHg	82 ± 9	86 ± 11	NS
MAP, mmHg	101 ± 12	106 ± 12	0.045
Fasting glucose, mmol/L	5.7 (4.9–7.9)	4.8 (4.6–5.2)	0.022
Insulin, *μ*U/mL^b^	7.60 (5.85–18.89)	9.88 (6.12–13.50)	NS
HOMA-IR^b^	1.68 (1.20–4.44)	1.89 (1.32–3.12)	NS
CRP, mg/L	8.81 (2.19–24.3)	4.86 (3.06–9.82)	NS
ADMA, *μ*mol/L	0.86 ± 0.22	0.72 ± 0.16	0.022
Ca, mmol/L	2.18 ± 0.16	2.25 ± 0.27	NS
Pi, mmol/L	1.50 (1.34–1.79)	1.41 (1.21–1.86)	NS
Ca × Pi, mmol^2^/L^2^	3.15 (2.88–3.77)	3.51 (2.86–3.91)	NS
iPTH, pg/mL	213 (179–512)	290 (230–428)	NS
OPG, pmol/L	9.36 (5.93–12.38)	5.10 (2.40–7.70)	0.014
OPN, ng/mL	310 (208–559)	304 (217–377)	NS
OC, ng/mL	41.7 (31.7–69.5)	42.0 (23.9–56.0)	NS
CCA-IMT, mm	0.98 ± 0.13	0.86 ± 0.14	0.006
All-cause mortality, *N* (%)	16 (47%)	3 (12%)	0.004
Cardiovascular mortality, *N* (%)	13 (38%)	3 (12%)	0.025

^a^Data for the group of HD patients only (21 patients with calcifications and 15 patients without calcifications).

^b^Data for nondiabetic patients only (16 patients with calcifications and 24 patients without calcifications).

**Table 2:** Differences in clinical criteria of the metabolic syndrome between patients with and without vascular calcifications as assessed by alizarin red staining of the radial artery.

	Patients with vascular calcifications (grades 1–4, *N* = 34)	Patients without vascular calcifications (grades 0, *N* = 25)	*P*
BMI > 25 kg/m^2^ (overweight or obesity), *N* (%)	18 (53%)	14 (56%)	NS
Fasting glucose > 5.6 mmol/L (IFG or diabetes), *N* (%)	21 (62%)	4 (16%)	0.0004
Hypertension, *N* (%)	28 (82%)	23 (92%)	NS
Low HDL^a^, *N* (%)	13 (38%)	7 (28%)	NS
High TG (>1.7 mmol/L), *N* (%)	15 (44%)	12 (48%)	NS
Three or more of above criteria present, *N* (%)	20 (59%)	9 (36%)	NS

^a^Low HDL cholesterol (<1.0 mmol/L in men, <1.3 mmol/L in women).

**Table 3:** Multiple logistic regression models to study the associations of the selected variables with vascular calcifications as assessed by alizarin red staining of the radial artery.

	Model 1^a^		Model 2^b^		Model 3^a^	
	OR (95% CI)	*P*	OR (95% CI)	*P*	OR (95% CI)	*P*
Age, years	1.05 (0.99–1.10)	NS	1.05 (0.99–1.11)	NS	1.00 (0.93–1.08)	NS
Fasting glucose > 5.6 mmol/L^c^	8.24 (1.66–40.9)	**0.008**	14.8 (1.68–130)	**0.012**	23.8 (1.84–309)	**0.012**
MAP, mmHg	—	—	—	—	0.93 (0.86–1.02)	NS
ADMA, 0.1 *μ*mol/L	—	—	—	—	1.66 (0.70–3.92)	NS
OPG, pmol/L	—	—	—	—	1.16 (0.92–1.45)	NS

Odds ratios for positive staining are presented.

^a^Adjusted for gender, dialysis status of patients, and Ca × Pi.

^b^Adjusted for the parameters of the metabolic syndrome: low HDL cholesterol (<1.0 mmol/L in men, <1.3 mmol/L in women), high triglycerides (>1.7 mmol/L), and high BMI (≥25 kg/m^2^), as well as hypertension, CRP, gender, dialysis status of patients, and Ca × Pi.

^c^Includes patients with IFG and patients with diabetes.

Bold font: statically significant data.
